# The Real-World Impact of App-Based Mindfulness on Headspace Members With Moderate and Severe Perceived Stress: Observational Study

**DOI:** 10.2196/52968

**Published:** 2024-03-04

**Authors:** Christine Callahan, Justin Kimber, Emily Hu, Leah Tanner, Sarah Kunkle

**Affiliations:** 1 Headspace, Santa Monica, CA, United States

**Keywords:** digital mental health, meditation, real word evidence, mental health, app, application, stress, observational study, depression, anxiety, tool, tools, psychological, psychological symptom, engagement, stress reduction, PSS-10

## Abstract

**Background:**

Perceived stress in the United States has drastically increased since the COVID-19 pandemic and is associated with negative mental health outcomes such as depression and anxiety. Digital mental health (DMH) interventions are efficacious tools to address negative mental health outcomes and have helped reduce the severity of psychological symptoms, such as anxiety, depression, and perceived stress, compared to waitlist controls. Although DMH tools have been studied in controlled settings, less is known about the real-world evidence of such interventions.

**Objective:**

This study aimed to (1) characterize patterns in baseline perceived stress and changes in perceived stress among Headspace members with moderate and severe baseline perceived stress and (2) examine associations between engagement with Headspace content and changes in perceived stress (ie, evaluate whether there is a dose-response relationship).

**Methods:**

We evaluated real-world perceived stress and engagement data at 2 time points among Headspace app members with baseline moderate and severe perceived stress. Perceived stress was measured using the Perceived Stress Scale (PSS-10) and engagement using active days and active minutes engaged with Headspace as well as the number of user sessions. Descriptive statistics were computed for all variables. Correlations between baseline and follow-up scores, percent change in PSS-10 scores, days between PSS-10 use, active days, active days per week, active minutes, active minutes per day, sessions, and sessions per week were evaluated. We used *t* tests to investigate differences in the abovementioned parameters between (1) participants who did and those who did not see improvements in PSS-10 scores (yes vs no improvement) and (2) participants who saw ≥30% improvement versus those who saw a <30% improvement in PSS-10 scores.

**Results:**

Overall, 21,088 Headspace members were included in these analyses. On average, members saw a 23.52% decrease in PSS-10 scores from baseline to follow-up. On average, members had 2.42 (SD 1.76) active days per week and 25.89 (SD 33.40) active minutes per day, and completed 7.11 (SD 8.34) sessions per week. *t* tests suggest that members who saw improvements in PSS-10 scores from baseline to follow-up had significantly higher baseline PSS-10 scores (Cohen *d*=0.56), more active days per week (Cohen *d*=0.33), and more sessions per week (Cohen *d*=0.27) than those who did not see improvements in PSS-10 scores (all *P*<.001). Additional *t* tests suggest that members with ≥30% improvement in PSS-10 scores had significantly higher baseline PSS-10 scores (Cohen *d*=0.35), more active days per week (Cohen *d*=0.36), and more sessions per week (Cohen *d*=0.31) than those with a >30% improvement (all *P*<.001).

**Conclusions:**

Real-world use of Headspace is associated with decreased perceived stress. Furthermore, data suggest that more engagement, specifically weekly active days and sessions, is associated with a greater likelihood of stress reduction.

## Introduction

Stress in the United States has increased, with significant impacts from the COVID-19 pandemic. While the pandemic itself caused a drastic increase in stress from 2019 to 2020 [1], stress levels still remain high and continue to impact the majority of Americans [2]. Higher perceived stress is associated with poorer mental health outcomes such as depression and anxiety [3]. Further meta-analytic research indicates small to medium effects of the relationship between perceived stress and mental health outcomes, suggesting that peoples’ appraisal of stressful situations in their lives might be a predictor of mental health outcomes [4,5]. Higher perceived stress also has economic costs, and studies have estimated that perceived stress, primarily work-related perceived stress, accounts for US $221 million to US $187 billion in both direct (eg, medical) and indirect costs (eg, absenteeism, burnout, and decreased productivity) [6]. This is important in the larger context of mental health costs and outcomes and how people manage stressors daily in their lives. Digital mental health (DMH) interventions may provide an accessible, scalable way to mitigate perceived stress.

DMH interventions are scalable and accessible; incorporate evidence-based practices (ie, mindfulness meditation and cognitive behavioral therapy techniques); are efficacious for a range of mental health concerns including anxiety, depression, and posttraumatic stress disorder [3]; and have shown to be effective in a wide range of populations with all levels of mental health concerns (ie, mild, moderate, and severe), including college students, employees, graduate trainees, and rural communities [7-9]. Studies suggest that digital mindfulness-based interventions significantly reduce perceived stress [10-12]. Specifically, a randomized controlled trial evaluated an app-based mindfulness tool among individuals with moderate and severe baseline perceived stress and suggested a 30.12% decrease in perceived stress from baseline to 8 weeks (intervention completion), with these reductions retained at 12-week follow-up (a 31.24% decrease) [12]. In addition to randomized controlled trials, current meta-analytic and systematic review data suggest medium effect sizes for DMH interventions for perceived stress from baseline to post intervention [4,5]. Beyond clinical outcomes, DMH interventions improve access to mental health care and provide individuals with effective, cost-effective care readily available via a mobile app or website.

While DMH interventions in clinical trials have been shown to be effective, less is known about their use in real-world settings. Real-world evidence builds upon clinical trials to improve our understanding of an intervention’s efficacy in a person’s daily functioning, providing data on the effectiveness and accessibility of DMH products as well as the external validity of interventions. As DMH interventions are delivered via a mobile device or computer to use within one’s own environments, real-world evidence highlights intervention effectiveness outside of controlled settings. As such, there is an increasing need to understand the real-world effectiveness of widely used commercial apps and DMH interventions that help improve mental health outcomes for the overall population.

Headspace is a popular and evidence-based DMH platform with over 100 million downloads and supported by >50 published peer-reviewed studies. The Headspace app offers a range of services, most notably to this study, mindfulness and meditation-based content that teaches coping strategies to manage daily stressors. Previous clinical trials on the efficacy of the Headspace app show evidence of improved mindfulness, focus, stress, sleep quality, burnout, resilience, anxiety, depression, and quality of life. Given the reach and scale of Headspace’s membership, there is an opportunity to better understand real-world outcomes beyond clinical trials. As such, this study aims to use real-world data to evaluate perceived stress among Headspace members. To accomplish this goal, this study’s aims are to (1) characterize patterns in baseline perceived stress and changes in perceived stress among Headspace members with moderate and severe baseline perceived stress and (2) examine associations between engagement with Headspace content and changes in perceived stress (ie, to evaluate if there is a dose-response relationship).

## Methods

### Study Design and Participants

**Figure 1.**
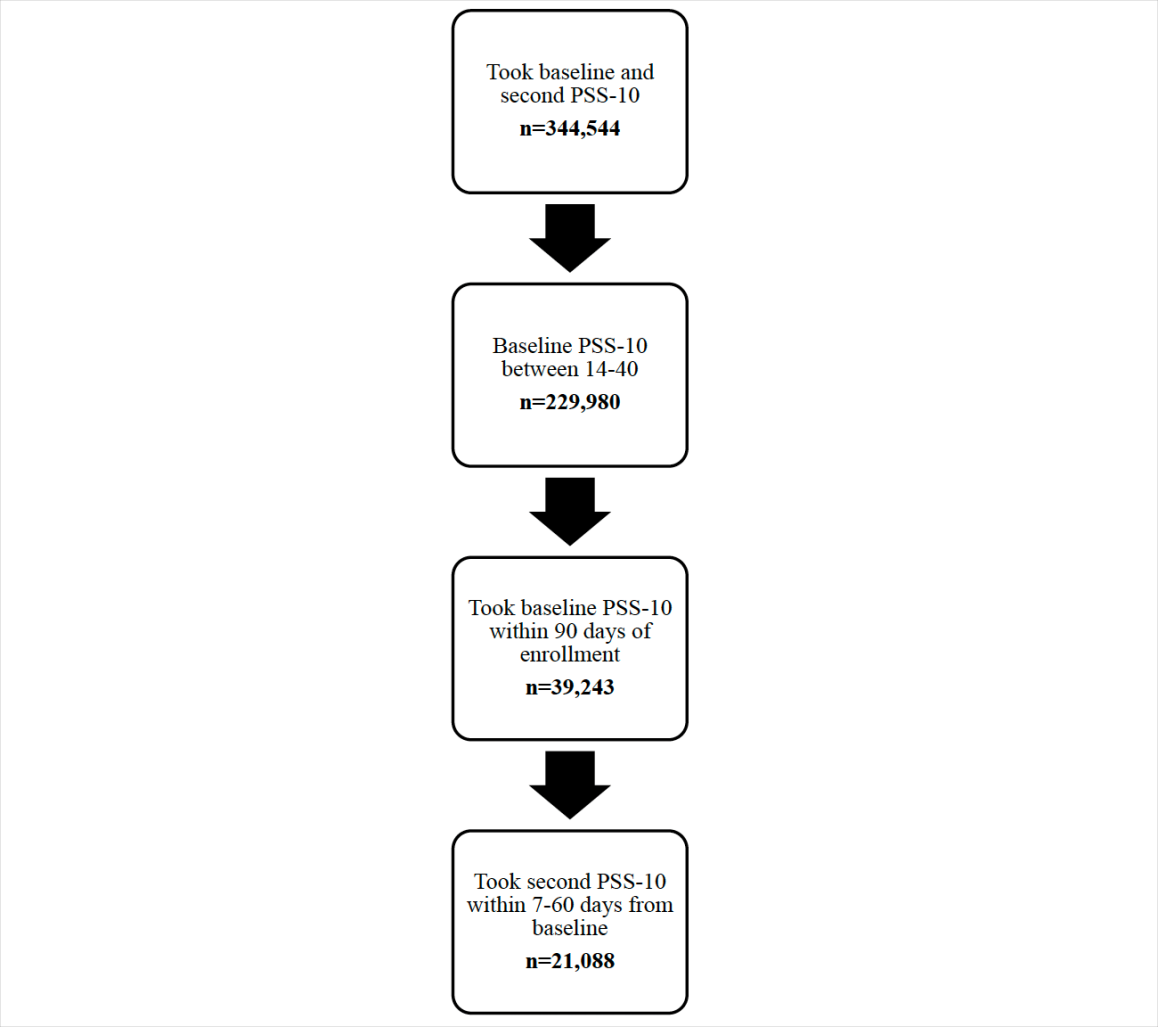
Sample size filter for the current study. PSS-10: Perceived Stress Scale.

This real-world observational study examined perceived stress among Headspace members. This study followed the STROBE (Strengthening the Reporting of Observational Studies in Epidemiology) framework for reporting observational studies [13]. Individuals were included in this study if they enrolled on Headspace between March 2020 and January 2023, completed a baseline and follow-up perceived stress questionnaire (the Perceived Stress Scale [PSS-10]), completed the baseline PSS-10 within 90 days of enrollment, completed the follow-up PSS-10 at >7 and <60 days from the baseline, and reported moderate or severe perceived stress levels at baseline (Figure 1).

### The Headspace App

**Figure 2.**
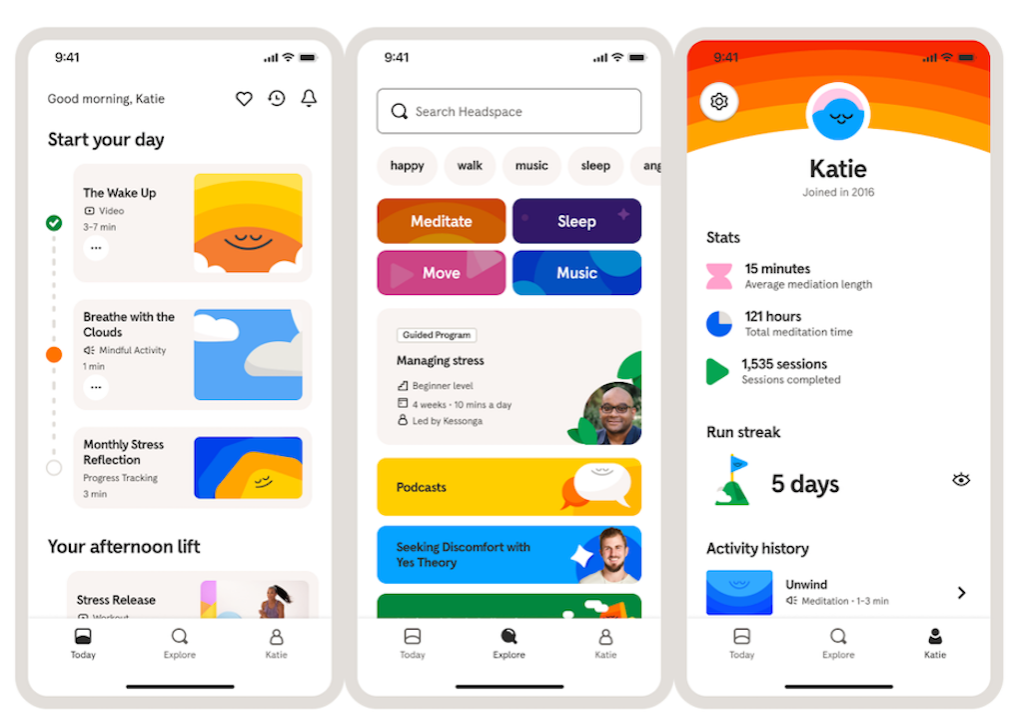
The “Today,” “Explore,” and “Profile” tabs in the Headspace app shown from left to right.

Study participants had access to all Headspace app offerings and used the app at their own discretion. The Headspace app includes evidence-based, expert-led content including guided mindfulness, meditation with animated guided videos, progressive muscle relaxation, psychoeducation, and gratitude exercises. Mindfulness content ranges from content for general self-care to that specific to mental health disorders such as anxiety, stress, and sleep disorders. When participants open the Headspace app, they are first directed to the “Today” tab, which contains personalized, daily content recommendations to encourage health habit formation throughout the day. Content on the “Today” tab includes a breathing exercise, educational video, and 3 meditations (1 each for the morning, afternoon, and evening). All other content can be found in the “Explore” tab, which includes a search bar; content organized into large categories of meditate, sleep, move, and music at the top; and as they scroll further, more specific categories and courses such as Beginning Meditation, Mindfulness at Work, Mindful Eating, etc. Finally, all participants have a personalized “Profile” tab, which tracks activity history and overall app statistics (minutes meditated, sessions completed, and days in a row of content engagement). Study participants had access to all Headspace app offerings and navigated the app to choose their own content (Figure 2).

### Ethical Considerations

This study represents a secondary analysis of preexisting deidentified data [14]. The study team does not have access to the participants’ identifying information and will not contact the participants. Therefore, in accordance with the US Department of Health and Human Services’ regulations (45 CRF 46.104), this study is deemed exempt from institutional review board oversight and informed consent. Participants are notified through the Headspace terms of service that their deidentified data may be used for research.

### Measures

Perceived stress was measured using the PSS-10 [15]. Participants were prompted to complete the PSS-10 through the Headspace app and answered 10 questions about their perceived feelings and thoughts regarding stress with a 5-point Likert scale (0=never to 4=very often). Scores range from 0‐40, with higher scores indicating higher perceived stress. The scale has high test-retest reliability (Cronbach α=.85). PSS-10 scores were categorized to indicate low (<14), moderate (14-26), and high (27-40) levels of perceived stress [15]. Those with low levels of perceived stress at baseline were excluded from this study. Study participants completed a baseline and follow-up PSS-10. Total scores for each time point were reported, along with the raw change in PSS-10 score and percent change in PSS-10 score. Participants completed the PSS-10 within the Headspace app, and final scores were extracted directly from the app. Scores were calculated and extracted only for those who completed all questions on the PSS-10 (ie, incomplete data were excluded from this study).

Engagement was assessed using active days and minutes engaged with Headspace as well as the number of sessions started. Additionally, ratios for active days per week, active minutes per day, and sessions per week were calculated to further define engagement. Engagement data are tracked for all members within the Headspace app and were extracted directly from the app.

### Statistical Analysis

Means, SDs, medians, and 95% CIs were computed for baseline and follow-up PSS-10 scores, raw change in PSS-10 score, percent change in PSS-10 score, days between PSS-10 assessments, active days, active days per week, active minutes, active minutes per day, sessions, and sessions per week. Frequencies and percentages were computed for baseline and follow-up PSS-10 scores (moderate vs high).

Mean percent change in PSS-10 scores was segmented out by active days per week, active minutes per day, and sessions per week to delineate the relationship between app engagement and percent change in PSS-10 score. Correlations between baseline and follow-up PSS-10 scores, percent change in PSS-10 score, days between PSS-10 assessments, active days, active days per week, active minutes, active minutes per day, sessions, and sessions per week were evaluated.

We used *t* tests to evaluate the differences in baseline PSS-10 scores, follow-up PSS-10 scores, percent change in PSS-10 score, active days, active days per week, active minutes, active minutes per day, sessions, and sessions per week between (1) those who saw and those who did not see improvements in PSS-10 scores (yes vs no improvement) and (2) those who saw a ≥30% improvement versus those who saw a <30% improvement. A 30% improvement threshold was chosen to coincide with a clinically meaningful change in perceived stress [16]. *P* values of <.05 were considered significant, and effect sizes were reported using the Cohen *d* to determine the magnitude of significance.

## Results

### Sample and Perceived Stress Descriptives

Overall, 344,544 Headspace members completed 2 PSS-10s between March 2020 and January 2023. Of them, 21,088 met this study’s inclusion criteria and were included as study participants (Figure 1).

At baseline, 15,127 (71.73%) participants reported moderate levels of perceived stress and 5961 (28.27%) reported severe levels of perceived stress with a mean baseline PSS-10 score of 23.14 (SD 5.69). On average, members completed their follow-up PSS-10 33.25 (SD 11.17) days after their first PSS-10 with a mean follow-up PSS-10 score of 20.41 (SD 6.47). At follow-up, 2878 (13.56%) participants reported mild to moderate levels of perceived stress, 14,376 (68.17%) reported moderate levels, and 3834 (18.18%) reported high levels. On average, members saw a 23.52% decrease in PSS-10 scores from baseline to follow-up, and 13,692 (64.93%) participants saw a decrease in their PSS-10 score (Table 1).

**Table 1.** Descriptive statistics for engagement and primary outcomes.

Parameters		Mean (SD)	Median	95% CI
**PSS-10^a^**				
	Baseline	23.14 (5.69)	23	23.06 to 23.21
	Follow-up	20.41 (6.47)	20	20.33 to 20.50
	Raw change in the PSS-10 score	–2.72 (6.06)	–3	–2.80 to –2.64
	Percent change in the PSS-10 score	–23.52 (62.60)	–13.04	–24.37 to –22.68
	Days between PSS-10 assessments	33.25 (11.17)	31	33.10 to 33.40
**Engagement metrics**				
	Active days	18.04 (11.42)	17	17.89 to 18.20
	Active days per week	2.42 (1.76)	2.01	2.39 to 2.44
	Active minutes	547.4 (1004.81)	281.95	533.83 to 560.96
	Active minutes per day	25.89 (33.40)	16.68	25.44 to 26.34
	Sessions	49.73 (48.14)	35	49.08 to 50.38
	Sessions per week	7.11 (8.34)	4.33	7.00 to 7.23

a PSS-10: Perceived Stress Scale.

## Engagement

On average, members engaged with Headspace content for 18.08 (SD 11.42) days and 547.40 (SD 1004.81) minutes and started 49.73 (SD 48.14) sessions. On average, members had 2.42 (SD 1.76) active days per week, 25.89 (SD 33.40) active minutes per day, and completed 7.11 (SD 8.34) sessions per week (Table 1). The percent change in PSS-10 score by active days per week, active minutes per day, and sessions per week are presented in Table 2, with data suggesting peak changes in PSS-10 scores at 7 active days per week, 11‐15 active minutes per day, and 19‐20 sessions per week.

**Table 2.** Mean percent change in PSS-10^a^ scores by active days per week, active minutes per day, and sessions per week.

		Mean percent change in PSS-10 score
**Active days per week**		
	1	–21.11
	2	–21.55
	3	–23.48
	4	–28.94
	5	–23.69
	6	–32.61
	7	–43.24
**Active minutes per day**		
	0-5	–19.86
	6-10	–21.33
	11-15	–24.41
	16-20	–24.26
	21-25	–23.84
	26-30	–25.65
	>30	–21.33
**Sessions per week**		
	1-2	–17.18
	2-4	–21.75
	5-6	–25.88
	7-8	–23.25
	9-10	–27.56
	11-12	–27.89
	13-14	–29.64
	15-16	–32.21
	17-18	–35.94
	19-20	–41.71
	>20	–25.39

a PSS-10: Perceived Stress Scale.

### Engagement and Perceived Stress

Correlations between engagement and perceived stress are reported in Table 3. The results of *t* tests investigating the association between engagement and perceived stress are reported in Table 4. Participants who demonstrated an improvement in perceived stress had significantly higher baseline PSS-10 scores (*t*_16,575_=–40.08; *P*<.001; Cohen *d*=0.56) and significantly more active days (*t*_14,774_=–9.00; *P*<.001; Cohen *d*=0.13), active days per week (*t*_17,152_=–24.18; *P*<.001; Cohen *d*=0.33), sessions (*t*_16,515_=–11.18; *P*<.001; Cohen *d*=0.16), and sessions per week (*t*_19,025_=–20.53; *P*<.001; Cohen *d*=0.27) than those who did not demonstrate an improvement.

Participants with a ≥30% improvement in perceived stress had significantly higher baseline PSS-10 scores (*t*_12,794_=–23.52 *P*<.001; Cohen *d*=0.35), more active days per week (*t*_11,401_=–23.10; *P*<.001; Cohen *d*=0.36), and more sessions per week (*t*_10,150_=–18.87; *P*<.001; Cohen *d*=0.31) than those with a <30% improvement. Although effect sizes suggest smaller relationships, *t* tests also suggest that participants with a ≥30% improvement in PSS-10 scores had significantly more active days (*t*_12,984_=–12.02; *P*<.001; Cohen *d*=0.17), more active minutes (*t*_13,569_=–2.61; *P*<.001; Cohen *d*=0.04), and more sessions (*t*_11,539_=–12.68; *P*<.001; Cohen *d*=0.19) than those with a <30% improvement in PSS-10 score.

**Table 3.** Pearson correlations between PSS-10^a^ scores and engagement outcomes.

		BaselinePSS-10 score	Follow-up PSS-10 score	Percent change in PSS-10 score	Days between PSS-10 assessments	Active days	Active days per week	Active minutes	Active minutes per day	Sessions	Sessions per week
**Baseline PSS-10 score**											
	*r*	N/A^b^	0.51	–0.14	–0.03	–0.12	0.05	0.01	0.04	–0.03	0.08
	*P* value	N/A	*<.001^c^*	*<.001*	*<.001*	*<.001*	*<.001*	.66	*<.001*	*<.001*	*<.001*
**Follow-up PSS-10 score**											
	*r*	0.51	N/A	0.51	0.03	–0.17	–0.13	–0.01	0.03	–0.11	–0.09
	*P* value	*<.001*	N/A	*<.001*	*<.001*	*<.001*	*<.001*	.05	*<.001*	*<.001*	*<.001*
**Percent change in PSS-10 score**											
	*r*	–0.14	0.51	N/A	0.03	–0.05	–0.12	–0.01	–0.01	–0.07	–0.10
	*P* value	*<.001*	*<.001*	N/A	*<.001*	*<.001*	*<.001*	.18	.97	*<.001*	*<.001*
**Days between PSS-10 assessments**											
	*r*	–0.03	0.03	0.03	N/A	0.33	–0.11	0.12	–0.01	0.16	–0.13
	*P* value	*<.001*	*<.001*	*<.001*	N/A	*<.001*	*<.001*	*<.001*	.12	*<.001*	*<.001*
**Active days**											
	*r*	–0.12	–0.17	–0.05	0.33	N/A	0.63	0.46	0.21	0.73	0.43
	*P* value	*<.001*	*<.001*	*<.001*	*<.001*	N/A	*<.001*	*<.001*	*<.001*	*<.001*	*<.001*
**Active days per week**											
	*r*	0.05	–0.13	–0.12	–0.11	0.63	N/A	0.31	0.19	0.58	0.78
	*P* value	*<.001*	*<.001*	*<.001*	*<.001*	*<.001*	N/A	*<.001*	*<.001*	*<.001*	*<.001*
**Active minutes**											
	*r*	0.01	−0.01	−0.01	0.12	0.46	0.31	N/A	0.87	0.52	0.36
	*P* value	.66	.05	.18	*<.001*	*<.001*	*<.001*	N/A	*<.001*	*<.001*	*<.001*
**Active minutes per day**											
	*r*	0.04	0.03	−0.01	−0.01	0.21	0.19	0.87	N/A	0.35	0.30
	*P* value	*<.001*	*<.001*	.97	.12	*<.001*	*<.001*	*<.001*	N/A	*<.001*	*<.001*
**Sessions**											
	*r*	–0.03	–0.11	–0.07	0.16	0.73	0.58	0.52	0.35	N/A	0.78
	*P* value	*<.001*	*<.001*	*<.001*	*<.001*	*<.001*	*<.001*	*<.001*	*<.001*	N/A	*<.001*
**Sessions per week**											
	*r*	0.08	–0.09	–0.10	–0.13	0.43	0.78	0.36	0.30	0.78	N/A
	*P* value	*<.001*	*<.001*	*<.001*	*<.001*	*<.001*	*<.001*	*<.001*	*<.001*	*<.001*	N/A

a PSS-10: Perceived Stress Scale.

b N/A: not applicable.

c Italicized values are significant at *P*<.05.

**Table 4.** Differences in PSS-10^a^ scores and engagement metrics between participants who saw an improvement in their PSS-10 score and those who did not and between participants who saw a ≥30% improvement in the PSS-10 score and those who saw a <30% improvement in their PSS-10 score.

	PSS-10 score improvement, mean (SD)	No PSS-10 score improvement, mean (SD)	95% CI	*t* test (*df*)	*P* value	Cohen *d*	≥30% improvement in PSS-10 score, mean (SD)	<30% improvement in PSS-10 score, mean (SD)	95% CI	*t* test (*df*)	*P* value	Cohen *d*
Baseline PSS-10 score	24.22 (5.69)	21.14 (5.12)	–3.23 to –2.93	–40.08 (16,576)	*<.001* ^b^	0.56	24.49 (5.59)	22.52 (5.63)	–2.12 to –1.80	–23.52 (14,364)	*<.001*	0.35
Follow-up PSS-10 score	18.16 (5.73)	24.60 (5.62)	6.28 to 6.60	78.87 (15,418)	*<.001*	1.13	15.13 (4.52)	22.81 (4.52)	7.54 to 7.83	104.68 (14,980)	*<.001*	1.42
Percent change in PSS-10 score	–43.42 (69.50)	13.32 (11.84)	55.55 to 57.94	93.07 (15,121)	*<.001*	1.01	–74.19 (90.34)	–0.53 (17.34)	71.45 to 75.85	65.60 (6682)	*<.001*	1.40
Total active days	18.57 (11.28)	17.07 (11.62)	–1.82 to –1.17	–9.00 (14,774)	*<.001*	0.13	19.43 (11.21)	17.41 (11.46)	–2.35 to –1.69	–12.02 (14,236)	*<.001*	0.17
Active days per week	2.62 (1.82)	2.04 (1.57)	–0.63 to –0.53	–24.18 (17,152)	*<.001*	0.33	2.85 (1.88)	2.22 (1.66)	–0.68 to –0.57	–23.10 (13,437)	*<.001*	0.36
Total active minutes	557.59 (969.71)	528.52 (1066.57)	–58.31 to 0.17	–1.95 (13,969)	.05	0.03	573.54 (956.53)	535.53 (1025.86)	–66.52 to −9.51	–2.61 (14,700)	*.01*	0.04
Active minutes per day	26.10 (32.33)	25.50 (35.29)	–1.56 to 0.38	–1.20 (14,062)	.23	0.02	26.25 (31.60)	25.73 (34.18)	–1.46 to 0.43	–1.07 (14,540)	.28	0.01
Total sessions	52.36 (49.64)	44.85 (44.85)	–8.83 to –6.20	–11.18 (16,515)	*<.001*	0.16	56.21 (51.62)	46.79 (46.18)	–10.87 to –7.96	–12.68 (13,549)	*<.001*	0.19
Sessions per week	7.90 (9.00)	5.65 (6.72)	–2.47 to –2.04	–20.53 (19,025)	*<.001*	0.27	8.88 (9.82)	6.31 (7.44)	–2.83 to –2.30	–18.87 (12,439)	*<.001*	0.31

a PSS-10: Perceived Stress Scale.

b Italicized values are significant at *P*<.05.

## Discussion

This study focused on using real-world data from Headspace members to evaluate changes in perceived stress and its association with app engagement. Our findings suggest that participants experienced a significant reduction in perceived stress scores and those who used Headspace more frequently experienced greater reductions in perceived stress. This study builds on prior clinical trials and provides real-world evidence supporting the use of Headspace to improve perceived stress.

On average, participants experienced a 23.52% reduction in perceived stress scores from baseline to the follow-up assessment (ie, approximately a 30-day period). Furthermore, 64.93% of participants saw a reduction in their PSS-10 scores from baseline to follow-up, suggesting that Headspace improved perceived stress for a majority of individuals. Shifts in perceived stress levels also provide evidence supporting the use of Headspace, as 10% of members who reported high levels of perceived stress at baseline no longer met those criteria at follow-up (ie, reported moderate or mild levels of perceived stress at follow-up). These shifts in individual perceived stress are important for self-management behaviors (eg, mindfulness or self-regulation) that are associated with improved patient well-being and mental health outcomes [17]. Thus, it is likely that shifts in perceived stress might be an important predictor of patients’ mental health outcomes. These findings are consistent with previous clinical research examining the impact of DMH interventions to improve perceived stress [10,18,19] and provide additional real-world evidence to support those claims.

We also identified relationships between app engagement and changes in perceived stress. Specifically, correlations suggest that active days per week and active sessions per week were associated with a higher percent change in PSS-10 scores. App-based mindfulness interventions of longer duration, such as those from 4 to 12 weeks, have shown improvements in not only mindfulness and perceived stress but also depression, anxiety, and overall well-being [11,20,21]. Few app-based mindfulness interventions are less than 4 weeks in duration, supporting the use of longer interventions. While this study revealed that a higher number of active days (total and per week) is associated with larger improvements in perceived stress, our data did not reveal a relationship between active minutes (total and per week) and percent change in perceived stress. These data support the findings of a previous study comparing 10 and 30 minutes of daily mindfulness, which reported no difference in mindfulness or psychological distress between the dosage groups [22]. This study’s findings, in addition to those of previous studies investigating mindfulness dosage, may suggest that active days are more important than total time spent practicing mindfulness or engaging with the app. Future research is necessary to further understand these relationships to more accurately suggest mindfulness dosage for clinical practice and research.

Identifying changes in perceived stress by active days per week, active minutes per day, and sessions per week provide more nuanced data informing the duration and dosage of mindfulness interventions. The largest percent change in perceived stress occurred with 7 active days per week; however, data show a sizable increase at 4 active days per week with a leveling off in those active for more days. We also note peak percent change in PSS-10 score at 11‐15 active minutes per day and 19‐20 sessions per week. These data indicate that consistent Headspace use results in more prominent improvements in perceived stress. Previous research has reported that the psychological status of approximately 25% of patients improved after 1 psychotherapy session, with steadier improvements occurring over 8 weeks [23]. This study’s findings indicate a similar pattern that emphasizes a shift in perceived stress from baseline to the second administration of the PSS-10 during the first 3 weeks of engaging with the Headspace platform.

Higher engagement, specifically more active days and sessions, was significantly associated with a higher likelihood of improving stress (both overall and ≥30%), suggesting that frequent engagement with the Headspace app might be related to improved outcomes. This is an important finding given the breadth of research on increased patient engagement with psychotherapy and improved mental health outcomes [24]. In particular, the effect sizes for active days per week and sessions per week of –0.33 and 0.27 in the group that saw improvements in perceived stress and the group that did not see an improvement and 0.36 and 0.31 in the group that saw a ≥30% improvement in perceived stress and the group that saw a <30% improvement are notable. These results build on previously reported correlations, suggesting that higher engagement days and a higher number of sessions are associated with a higher percent change in PSS-10 scores and provide further support for the intervention dosage, suggesting multiple bouts of mindfulness each day [22]. Previous research highlights positive mental health outcomes for people who establish consistent health behaviors. In light of higher costs and access to mental health care, these preliminary findings suggest that consistently engaging with Headspace may decrease perceived stress, thus supporting the use of DMH in real-world settings to accessibly improve outcomes.

### Strengths, Limitations, and Future Research

This study has several limitations and strengths. As this study was the first real-world study investigating the Headspace app, our data are largely focused on descriptive and group mean differences. While this information is important to establish overall benchmarks for this study, we are limited in the types of questions (eg, prospective) we can ask with this study’s format and recognize that the current statistical approach does not allow for causal inferences. Additionally, these data did not include the demographic information of Headspace members; therefore, we were unable to investigate the impact of demographics on the study outcomes. Future research should examine longitudinal data and changes in perceived stress, while accounting for the demographic characteristics of Headspace members and engagement factors. As a real-world evidence study, we were unable to identify specific programming used by members; we focused only on overall engagement outcomes. Future studies should include more in-depth engagement outcomes to better understand how certain programming is used and impacts perceived stress. Finally, data for this study were collected during the COVID-19 pandemic (March 2020 and January 2023). As noted, stress greatly increased among individuals in the United States during this time, which may have impacted PSS-10 scores (ie, increased scores). However, members were only included if they completed their 2 PSS-10s between 7 and 60 days, and the average number of days between PSS-10 assessments was approximately 1 month, suggesting that if members completed their baseline PSS-10 at the beginning of the pandemic, when stress levels were higher nationwide, they would have completed their follow-up at a similar time period within the pandemic.

A primary strength of the study is the methodology incorporated to understand changes in perceived stress and engagement on the platform. The use of a real-world evidence methodology often helps researchers and clinicians to observe the feasibility and generalizability of interventions in daily functioning. Given the current sample size, which is over 20,000 participants, it is evident that the Headspace platform provides benefits to people who frequently engage with the platform. Furthermore, this study’s large sample size also offers strong evidence for the generalizability of our findings in the real world. The large sample size coupled with the current methodology also allowed us to establish reliable findings for our current platform; therefore, establishing a foundational understanding of how much change is possible on the platform while understanding overall engagement trends.

### Conclusions

Our findings suggest that members using Headspace experienced significant reductions in perceived stress in a real-world setting. Furthermore, data suggest that members who engaged with the platform more regularly were more likely to experience improvements in perceived stress. This study is the first to provide real-world evidence of the DMH Headspace platform aimed to reduce participants’ perceived stress. Our results have implications for clinical practice, which include incorporating mediation and mental health psychoeducation as an adjunct to psychotherapy or as a preventive intervention to reduce stress.

## Abbreviations

DMH: digital mental health
PSS-10: Perceived Stress Scale
STROBE: Strengthening the Reporting of Observational Studies in Epidemiology
